# Herpes zoster diagnosis and treatment in relation to incident dementia: A population-based retrospective matched cohort study

**DOI:** 10.1371/journal.pone.0296957

**Published:** 2024-01-25

**Authors:** Sheila Weinmann, Andreea Rawlings, Padma Koppolu, A. Gabriela Rosales, Yolanda K. Prado, Mark A. Schmidt

**Affiliations:** Kaiser Permanente Center for Health Research, Kaiser Permanente Northwest, Portland, Oregon, United States of America; Wingate University, UNITED STATES

## Abstract

**Background:**

Evidence suggests that some infectious diseases, such as herpes zoster (HZ), are associated with elevated risk of subsequent dementia, while certain anti-viral medications are associated with lower risk. We sought to evaluate associations between HZ diagnosis and treatment with incident dementia in a large, retrospective matched cohort.

**Methods:**

Using ICD-9 and ICD-10 diagnosis codes in electronic medical records, we identified members of Kaiser Permanente Northwest age 50 and older from 2000–2019 with a HZ diagnosis during this period. A comparison group without HZ diagnosis was individually matched 3:1 on age at HZ diagnosis date (index date), sex, and membership length prior to index date. We excluded subjects with dementia diagnosed before the index date. Antiherpetic medication was identified using pharmacy fills 1 month before to 12 months after the index date. We employed survival analysis to examine the associations between dementia and HZ diagnosis and antiherpetic medication, adjusting multivariable models for demographic and clinical factors. We stratified on age and sex and conducted a sensitivity analysis with a 5-year lag period.

**Result:**

The study included 101,328 persons, 25,332 with HZ. Over a median follow-up of 4.8 years, 6,000 developed dementia. HZ diagnosis was not associated with higher hazard of dementia (hazard ratio (HR) = 0.99, 95% CI 0.93–1.05) in the primary analysis. Among persons with HZ diagnoses, the HR for receipt of any antiherpetic medication was 0.79 (95% CI 0.70–0.90) in univariate analysis and 0.88 (95% CI 0.77–1.00) after adjustment for demographic and clinical factors. Dementia was not associated with trends in duration of medication use or cumulative dose.

**Conclusions:**

We found little evidence for an association between HZ diagnosis and dementia overall. Antiherpetic medication prescribed around the time of HZ diagnosis was statistically associated with lower risk of subsequent dementia in some but not all analyses and subgroups.

## Introduction

Dementia, an irreversible, progressive brain disorder that affects cognition and memory and interferes with daily life, is a major public health concern and cause of death worldwide. Among U.S. residents age 65 and older, it affected an estimated 5.8 million people in 2020, and that number is projected to grow to about 13.9 million by 2060 [1]. Although advanced age is the most important risk factor for dementia, education and socioeconomic status; genetic factors, particularly the *apolipoprotein E (APOE) e4* allele; and a variety of health conditions are also risk factors [2]. Some studies suggest that infectious agents such as herpes simplex virus-1 (HSV1), herpes zoster (HZ), and *Chlamydia pneumonia* may also play a role though there is robust debate about this [3–5]. Effective treatment for dementia is lacking.

Herpes zoster (HZ) is caused by reactivation of the varicella-zoster virus, which remains latent in the ganglionic neurons after initial varicella infection. Reasons for the reactivation of the virus are not always clear, but immunosuppression due to aging, immunocompromising medical conditions, and pharmacologic treatment are known to be associated [6]. In the U.S. Pacific Northwest in 1997–2002, incidence of HZ was approximately 370 per 100,000 person-years overall, and incidence increased sharply after age 50 to more than 1,100 per 100,000 person-years in persons age 75 years and older [7].

Several recently published studies in international populations have suggested that HZ diagnosis is associated with elevated risk of subsequent dementia diagnosis with hazard ratios (HRs) ranging from 1.1 to 2.8 [8–10]. Additionally, among persons with HZ, the use of certain antiviral medications for HZ treatment was found to be associated with lower risk of dementia diagnosis with HRs of 0.55 and 0.79 [8, 10], leading to hope that prophylactic antiherpetic drugs might help prevent dementia onset. To examine these important questions, replication studies in other populations are needed. Therefore, our study evaluated the association between HZ diagnosis and its treatment in relation to incident dementia in the population of a large integrated health plan in the United States.

## Materials and methods

### Study design

We used a retrospective matched cohort study design to examine HZ diagnosis in relation to subsequent dementia diagnosis. Using common terminology for cohort studies, in this report we refer to HZ diagnosis as the ‘exposure’ and dementia diagnosis as the ‘outcome’. Subjects with an HZ diagnosis are classified as ‘exposed’. Among persons with HZ in this cohort, we also examined the association of anti-herpetic treatment with dementia.

The Kaiser Permanente Northwest (KPNW) Institutional Review Board first approved this study on 2/4/2020. The KPNW IRB was later absorbed by the Kaiser Permanente Interregional Institutional Review Board, which also approved this study, Protocol #1551499; the most recent approval date was 11/27/2023.

### Population

Study subjects were members of the KPNW integrated health plan who were 50 years of age or older between 1/1/2000 and 12/31/2019 inclusive. Subjects who were eligible at the study start date (1/1/2000) entered the cohort at that time, while other subjects entered the cohort when they reached the age of 50 or when they joined the health plan and were otherwise eligible for the study. Those who had dementia diagnoses before age 50 or before the beginning of the study period were excluded. We also excluded subjects with missing data on sex and persons who opted out of research studies.

### Data collection

Clinical data were drawn from diagnosis codes, procedure codes, and medication orders in electronic medical records on 6/29/2021. We abstracted electronic health record data and used ICD-9-CM and ICD10-CM codes (053.0X-053.9X, ICD-9-CM codes 053.2, 053.20, 053.21, 053.22, 053.29) to identify subjects with at least one HZ diagnosis code during the study period. We did not include codes for post-herpetic neuralgia because that diagnosis is more likely to be associated with prevalent HZ rather than incident HZ. We included codes for herpes zoster opthalmicus (HZO) in the overall HZ group and also analyzed HZO separately in a sub-analysis. The date of the first HZ diagnosis in the study period was considered the index date for persons exposed to HZ. In previous work, we validated the HZ ICD-9-CM codes in the KPNW population and found a positive predictive value of 86% in patients age 50 and older [7].

Each person with a HZ diagnosis was individually matched with replacement to three people without HZ as of the exposed person’s index date; this date was considered the index date for the matched unexposed subjects. Matching criteria included age (plus or minus 1 year), sex, and length of membership before the index date (plus or minus 1 year). We excluded exposed and unexposed subjects who were diagnosed with dementia before the index date, and those with less than one full year of membership before the index date.

For the study outcome, we identified persons with two or more outpatient or inpatient ICD-9-CM or ICD-10-CM dementia codes recorded in a 12-month period after the index date and before the end of the study period (12/31/2019), as recommended by Harding et al. [11]. The date of the first dementia code was used as the dementia diagnosis date. The codes used are presented in S1 Table. Potential confounders and effect modifiers evaluated included the following risk factors for dementia: age (continuous and stratified by age group); sex; race (Black, Asian, White); Hispanic ethnicity (yes/no); ever-tobacco-smoker (yes/no); depression; traumatic brain injury; alcohol use disorder; diabetes; blood disorder; cancer; hearing loss; heart failure; hyperlipidemia; hypertension; and stroke/transient ischemic attack (all yes/no). We used one or more ICD-9-CM code, ICD-10-CM code, or Current Procedural Terminology (CPT) code at any time before the index date to define each of these covariates. The variable for autoimmune disorder (yes/no) was computed using ICD-9-CM and ICD-10-CM codes for lupus, celiac disease, Sjogren’s syndrome, multiple sclerosis, polymyalgia rheumatica, ankylosing spondylitis, Type 1 diabetes, alopecia areata, vasculitis, and temporal arteritis. The variable for immunocompromised status (yes/no) was created using a list of ICD-9-CM and ICD-10-CM codes describing immunocompromising medical conditions [12].

We also evaluated indicators of medical care use, including number of general practice, specialty, and inpatient visits (not including emergency department visits) in the year before the index date. We collected data on HIV but did not use that variable in the analysis due to small numbers.

Antiherpetic medication orders were identified from 1 month before the index date to 12 months after the index date. For HZ-exposed persons, this was an attempt to capture all anti-herpetic medications prescribed for the HZ episode. To make it highly likely that the medication was prescribed for HZ, we required the first medication order to be within 30 days of the index date. Antivirals examined included acyclovir, famciclovir, ganciclovir, and valganciclovir. We constructed a variable for any anti-viral use (yes/no). For acyclovir, which constituted more than 99% of antiviral prescriptions, we calculated duration (number of days covered by filled prescriptions) and cumulative dose (total dose in all filled prescriptions) during the 13-month period of interest.

### Statistical analysis

The members of the matched cohort were followed from the index date to dementia diagnosis, HZ diagnosis, KPNW membership end date, date of death, or end of follow-up on 12/31/2019, whichever was earlier. We examined population characteristics overall and by exposure status. We used Cox regression models and Kaplan-Meier curves to examine the association between HZ diagnosis and dementia, overall and stratified by sex and by age group. All analyses were 2-tailed, with p-value <0.05 considered statistically significant. We used SAS version 9.4.

We first constructed univariate models, and then adjusted them for demographics [age (continuous); sex; race (Black, Asian, White); Hispanic ethnicity] and finally for demographic and clinical factors (age; sex; race; Hispanic ethnicity; general practice, specialty and inpatient visits; autoimmune disorder; immunosuppressed status; alcohol use disorder; blood disorder; cancer; depression; diabetes; hearing loss; heart failure; hyperlipidemia; hypertension; stroke/TIA; traumatic brain injury; and ever-smoker). Robust standard errors were used to account for subject correlation due to matching with replacement [13, 14].

Among subjects with HZ, we examined antiviral medication dispensings in relation to dementia diagnosis, and we compared demographic and clinical characteristics between persons with and without antiviral medication use. We also examined categories of dispensed medication duration (1–10 days vs. 11+ days) and cumulative dose (1–40,000 mg. vs. 40,001+ mg.) in relation to dementia. Since more than 99% of anti-herpetic prescriptions were for acyclovir, we restricted the duration and cumulative dose analyses to acyclovir only, excluding subjects with other antiherpetic medications from the analysis. Category cut-points for the duration and cumulative dose analyses were determined by the maximum recommended acyclovir prescription for an episode of HZ [15].

Medical scrutiny during HZ diagnosis and treatment could result in dementia ascertainment bias. Therefore, for both the HZ analysis and the antiviral analysis, we conducted sensitivity analyses excluding matched sets where either the exposed subject or the sole unexposed matched subject had less than 5 years of follow-up after the index date.

## Results

**Fig 1:**
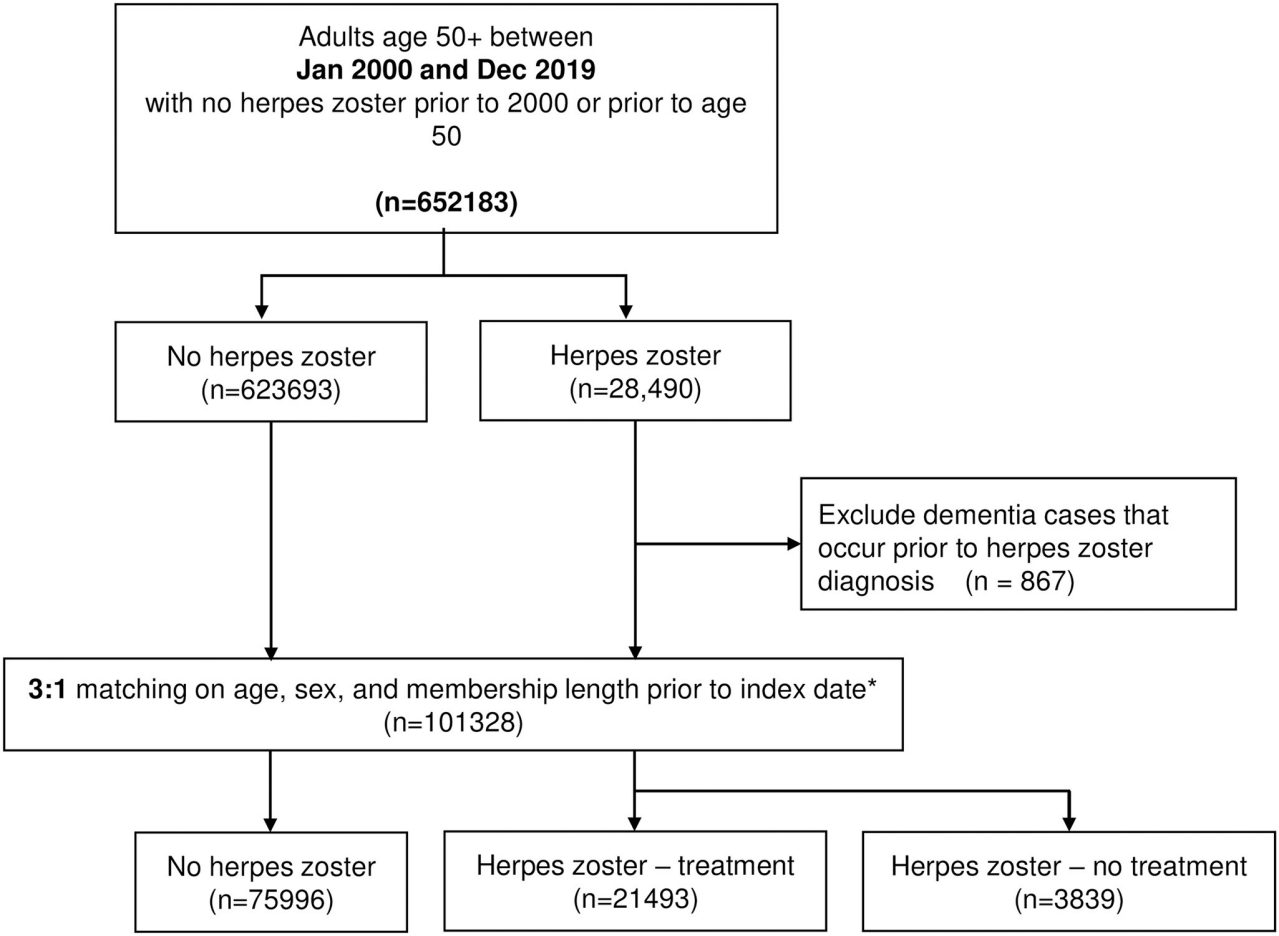
Study population flow diagram. *Excluding any potential controls with dementia diagnoses prior to the index date.

During the study period, in a health plan member population of 652,183 age-appropriate persons, we identified 28,490 persons with HZ diagnoses. After exclusions, we included in the study 25,332 eligible HZ-exposed subjects (Fig 1) and 75,996 unexposed subjects matched on age, sex, and health plan membership for a total of 101,328. Overall cumulative incidence of dementia in this matched cohort during the study period was 9.6 per 1,000 person-years (9.3–9.8). Demographics of the cohort are shown in Table 1. The population was primarily female (61%) and White (87%) with about half being age 65 years or older (48%). The exposed and unexposed groups were similar by race/ethnicity. Medical utilization was higher in the exposed group, as was immunosuppressed status, depression, and cancer history. Among the HZ-exposed subjects, 82% had at least one antiviral prescription, compared to 8% in the HZ-unexposed group. Among the HZ-exposed, compared to the antiviral recipients, those without an antiviral prescription were more likely to be men (41% vs. 38%), age 75 or older (23% vs. 20%) and to have had an inpatient stay in the study period (14% vs.11%).

**Table 1:** Demographic and clinical characteristics of HZ cases and matched unexposed subjects.

CHARACTERISTIC	UNEXPOSED	EXPOSED TO HZ	EXPOSED TO HZ WITH ANTIVIRAL USE	EXPOSED TO HZ WITHOUT ANTIVIRAL USE
	N = 75,996	N = 25,332	N = 20,842	N = 4,490
	N (%)	N (%)	N (%)	N (%)
SEX*				
Female	46,668 (61.4)	15,556 (61.4)	12,920 (62.0)	2,636 (58.7)
Male	29,328 (38.6)	9,776 (38.6)	7,922 (38.0)	1,854 (41.3)
RACE				
Asian	2,524 (3.3)	931 (3.7)	757 (3.6)	174 (3.9)
Black	1,649 (2.2)	426 (1.7)	357 (1.7)	69 (1.5)
Native Hawaiian/Pac. Isl.	386 (0.5)	141 (0.6)	118 (0.6)	23 (0.5)
American Indian/Alaskan Native	791 (1.0)	296 (1.2)	246 (1.2)	50 (1.1)
White	65,957 (86.8)	22,297 (88.0)	18,418 (88.4)	3,879 (86.4)
Other	298 (0.4)	103 (0.4)	84 (0.4)	19 (0.4)
Missing	4,391 (5.8)	1,138 (4.5)	862 (4.1)	276 (6.1)
HISPANIC ETHNICITY				
Yes	2,037 (2.7)	753 (3.0)	639 (3.1)	114 (2.5)
No	73,959 (97.3)	24,579 (97.0)	20,203 (96.9)	4,376 (97.5)
AGE IN YEARS*				
Median (25^th^, 75^th^)	64.0 (57.0, 73.0)	64.0 (57.0, 63.0)	64.0 (57.0, 72.0)	65.0 (58.0, 74.0)
50 ≤ Age < 65 years	39,338 (51.8)	13,097 (51.7)	10,931 (52.4)	2,166 (48.2)
65 ≤ Age < 75 years	20,883 (27.5)	6,981 (27.6)	5,707 (27.4)	1,274 (28.4)
Age ≥ 75 years	15,775 (20.8)	5,254 (20.7)	4,204 (20.2)	1,050 (23.4)
MEDICAL UTILIZATION IN YEAR BEFORE INDEX DATE				
General practice visits Median (25th, 75th)	5.0 (2.0, 9.0)	7.0 (4.0, 13.0)	8.0 (4.0, 13.0)	7.0 (4.0, 13.0)
General practice visits > = 5	40,803 (53.7)	18,028 (71.2)	14,963 (71.8)	3,065 (68.3)
Specialty visits Median (25th, 75th)	7.0 (3.0, 17.0)	10.0 (4.0, 24.0)	10.0 (4.0, 24.0)	10.0 (4.0, 24.0)
Specialty visits > = 7	40,866 (53.8)	16,320 (64.4)	13,470 (64.6)	2,850 (63.5)
Any inpatient stay during study period	5,829 (7.7)	2,967 (11.7)	2,318 (11.1)	649 (14.5)
COMORBIDITIES				
Hypertension	41,039 (54.0)	13,519 (53.4)	11,025 (52.9)	2,494 (55.5)
Diabetes	19,251 (25.3)	6,734 (26.6)	5,509 (26.4)	1,225 (27.3)
Stroke / TIA	7,715 (10.2)	2,656 (10.5)	2,124 (10.2)	532 (11.8)
Hearing loss	18,794 (24.7)	6,252 (24.7)	5,182 (24.9)	1,070 (23.8)
Depression	24,139 (31.8)	8,483 (33.5)	7,051 (33.8)	1,432 (31.9)
Immunosuppression	10,232 (13.5)	4,276 (16.9)	3,578 (17.2)	698 (15.5)
Cancer	10,745 (14.1)	4,048 (16.0)	3,306 (15.9)	742 (16.5)
Heart failure	5,827 (7.7)	2,199 (8.7)	1,736 (8.3)	463 (10.3)
Autoimmune disorder	2,692 (3.5)	1,231 (4.9)	1,030 (4.9)	201 (4.5)
Alcohol use disorder	5,015 (6.6)	1,569 (6.2)	1,330 (6.2)	239 (6.2)
Blood disorder	882 (1.2)	430 (1.7)	358 (1.7)	72 (1.6)
Traumatic brain injury	2,560 (3.4)	838 (3.3)	677 (3.2)	161 (3.6)
Hyperlipidemia	35,194 (46.3)	11,563 (45.7)	9,457 (45.4)	2,106 (46.9)
EVER-SMOKER				
Yes	35,642 (46.9)	12,197 (48.2)	10,014 (48.0)	2,183 (48.6)
No	40,354 (53.1)	13,135 (51.9)	10,828 (52.0)	2,307 (51.4)
ANTIVIRAL USE				
Yes	6,004 (7.9)	20,842 (82.3)	20,842 (100.0)	NA
No	69,992 (92.1)	4,490 (17.7)	NA	4,490 (100.0)
DEVELOPED DEMENTIA DURING FOLLOW-UP				
Yes	4,534 (6.0)	1,466 (5.8)	1,164 (5.6)	302 (6.7)
No	71.462 (94.0)	23,866 (94.2)	19,678 (94.4)	4,188 (93.3)
Cumulative dementia incidence per 1,000 person-years (95% CI)	9.6 (9.3–9.9)	9.4 (8.9–9.9)	8.9 (8.5–9.5)	11.4 (10.2–12.7)
LENGTH OF FOLLOW-UP (YEARS)*				
Mean±SD	6.2±5.0	6.2±5.0	6.2±5.0	5.9±5.0
Median (25th, 75th), All subjects	4.9 (2.0, 9.0)	4.9 (2.0, 9.0)	4.9 (2.0, 9.5)	4.6 (1.8, 9.0)
Median (25th, 75th), 50 ≤ Age < 65	5.1 (2.0, 10.0)	5.2 (2.0, 10.1)	5.2 (2.1, 10.2)	5.0 (1.8, 9.6)
Median (25th, 75th), 65 ≤ Age < 75	5.4 (2.3, 10.1)	5.2 (2.1, 9.8)	5.2 (2.2, 9.8)	5.0 (2.0, 9.8)
Median (25th, 75th), Age ≥ 75	4.2 (1.9, 7.7)	3.9 (1.7, 7.5)	4.0 (1.8, 7.7)	3.6 (1.4, 6.8)

* Mean difference (pooled standard deviation) in matching variables between HZ exposed and unexposed is as follows: age -0.0228 (SD 10.3179), follow-up days -0.0110 (12.1771), gender 0 (0.4868).

### Primary analyses

Median length of follow-up after the index date was similar at 4.9 years in HZ exposed and unexposed subjects. During the follow-up period, 6,000 subjects developed dementia, including 5.8 percent of HZ-exposed subjects and 6.0 percent of unexposed subjects. After adjustment for covariates, HZ diagnosis was not associated with higher hazard of dementia (HR = 0.99, 95% CI 0.93–1.05) (Table 2). We found no difference in risk by sex or age.

**Table 2:** Herpes zoster diagnosis in relation to hazard of dementia, overall and by age group and sex.

Variable	No. subjects with dementia/total subjects	HR (95% CI)
Exposed–univariate model	6,000/101,328	0.98 (0.92–1.04)
Exposed–adjusted for demographics and health care utilization*	6,000/101,328	0.95 (0.90–1.01)
Exposed–fully adjusted model**	6,000/101,328	0.99 (0.93–1.05)
50 ≤ Age < 65 years**	592/52,435	1.05 (0.87–1.27)
65 ≤ Age < 75 years**	1,828/27,864	1.02 (0.91–1.1)
Age ≥ 75 years**	3,580/21,029	0.97 (0.90–1.05)
Female**	3,851/62,224	1.02 (0.95–1.10)
Male**	2,149/39,104	0.92 (0.83–1.02)

* Adjusted for age (continuous); gender; race (Black, Asian, White); Hispanic ethnicity; general practice, specialty, and inpatient visits

** Adjusted for age (continuous); gender; race (Black, Asian, White); Hispanic ethnicity; general practice, specialty, and inpatient visits; autoimmune disorder; immunosuppression status; alcohol use disorder; blood disorder; cancer; depression; diabetes; hearing loss; heart failure; hyperlipidemia; hypertension; stroke/TIA; traumatic brain injury; and ever-smoker

When we examined the 338 HZO subjects and their unexposed matches separately, we obtained an adjusted HR of 1.14 (95% CI 0.79–1.65).

Among persons with HZ, receipt of any antiherpetic medication was associated with lower hazard of dementia in the univariate model and also in a model adjusted for demographics and health care utilization. After additional adjustment for multiple covariables, the overall HR was 0.88 (95% CI 0.77–1.00). We found only minor differences in the HR based on sex or age (Table 3).

**Table 3:** Anti-herpetic medication use* in relation to hazard of dementia among subjects with herpes zoster diagnoses, overall and by age group and sex.

Variable	No. subjects with dementia/total subjects	HR (95% CI)
Any antiviral–univariate model	1,466/25,332	0.79 (0.70–0.90)
Any antiviral adjusted for demographics and health care utilization**	1,466/25,332	0.86 (0.75–0.98)
Anti-viral–fully adjusted model***	1,466/25,332	0.88 (0.77–1.00)
50 ≤ Age < 65 years**	157/13,097	0.85 (0.56–1.29)
65 ≤ Age < 75 years**	452/6,981	0.89 (0.70–1.12)
Age ≥ 75 years**	857 /5,254	0.83 (0.70–0.97)
Female***	965/15,556	0.91 (0.77–1.08)
Male***	501/9,776	0.81 (0.65–1.01)

* First prescription from 1 month before HZ diagnosis date through 1 month after HZ diagnosis date. Includes acyclovir, famciclovir, ganciclovir, and valganciclovir.

** Adjusted for age (continuous); gender; race (Black, Asian, White); Hispanic ethnicity; general practice, specialty, and inpatient visits

*** Adjusted for age (continuous); gender; race (Black, Asian, White); Hispanic ethnicity; general practice, specialty, and inpatient visits; autoimmune disorder; immunosuppression status; alcohol use disorder; blood disorder; cancer; depression; diabetes; hearing loss; heart failure; hyperlipidemia; hypertension; stroke/TIA; traumatic brain injury; and ever-smoker

Among those with HZ and acyclovir dispensings, the median (25^th^, 75th) duration of use was 7 (7, 10) days, and the median cumulative dose was 28,000 (16,000, 72,000) mg. Mean (SD) duration of use was 17.6 (39.4) days, and mean (SD) cumulative dose was 41,139 (43,800) mg. When compared with no acyclovir use, there were no evident trends in dementia risk across categories of acyclovir duration or cumulative dose overall or in any age group. In the duration analysis including all HZ-exposed subjects, compared to no acyclovir use, the adjusted HR for a filled prescription for 1 to 10 days was 0.86 (95% CI 0.75–0.99) and the HR for 11 days or more was 0.92 (95% CI 0.78–1.09). For cumulative dose, compared to no acyclovir use, the adjusted HR for a filled prescription for 1 to 40,000 mg was 0.87 (95% CI 0.76–0.997) while the HR for 40,001 mg or more was 0.92 (95% CI 0.75–1.12).

### Sensitivity analyses

When we excluded subjects without at least 5 years of follow-up after the HZ diagnosis date, the HR for the association between HZ and dementia increased slightly to 1.07 (95% CI 0.99–1.17). HRs for the sex and age group subcategories were each a bit higher than before. The HR for women was 1.12 (95% CI 1.01–1.24) in contrast to the null HR for men (HR = 0.99, 95% CI 0.86–1.15) (S2 Table). The HR for the HZO subgroup was 0.84 (95% CI 0.49–1.45).

When we excluded subjects without at least 5 years of follow-up, results for the antiviral/dementia analysis were quite similar to the original results overall and for the sex and age subcategories.

## Discussion

In our population-based retrospective cohort study of over 25,000 persons with HZ and a matched comparison sample of over 75,000 persons without HZ, we found no evidence for a positive association between HZ diagnosis and subsequent dementia in the primary analysis. The slightly higher HRs in the sensitivity analysis including only dementia diagnosed at least 5 years after HZ diagnosis may be due to the dementia outcome taking a few years to develop and be diagnosed. Adjustment for covariates affected the HRs only slightly.

Among those with HZ diagnoses, antiherpetic medications prescribed around the time of the diagnosis were not statistically associated with lower risk of dementia after adjustment for potential confounders. For this analysis, we used as the reference group the relatively small proportion of the HZ-exposed subjects who did not have antiherpetic treatment (18%); this could be problematic, as the untreated group may have differed from the treated group in ways we were unable to measure. We do know that the untreated group was slightly older than the treated group and a slightly higher percentage had a history of hospitalization within one year prior to the index date. However, we adjusted for age, health care utilization, and a variety of other dementia risk factors in the multivariable models.

We did not observe a trend with either duration or cumulative dose of acyclovir in relation to dementia diagnosis. For this analysis, we created two categories of acyclovir duration and cumulative dose with the lower group representing the standard HZ treatment regimen (up to 10 days duration and up to 40,000 mg of acyclovir) and the higher group representing treatment days and cumulative dose above the standard amount. For both the duration and cumulative dose analyses, each of the two medication groups was compared to the no-treatment reference group. We also examined possible trends in the age subgroups. Though trends with duration or cumulative dose were not evident, we did observe a statistically significant lower risk of dementia when the standard HZ dose and duration treatment regimen was prescribed [HRs of 0.86 (95% CI 0.75–0.99) and 0.87 (95% CI 0.76–0.997) respectively] compared to the no treatment reference group.

Our decision to examine antiviral use only among those with a contemporaneous HZ diagnosis was intended to minimize possible confounding by indication, since the medication was most likely given for HZ and not for another condition that might have a different association with dementia. To avoid differential follow-up time for capturing possible medication use in subjects with and without eventual dementia diagnoses, we restricted the ascertainment period for antiherpetic drugs to the month directly before and the 12 months directly after the HZ diagnosis, with first prescription within one month of HZ diagnosis. Some subjects likely had subsequent antiviral use that we did not capture in our analysis. However, the median treatment duration in our acyclovir analysis was 7 days, which was not dissimilar to the 12-day median treatment period in the Chen et al. study, [8] which followed patients for a median of 6 years.

Population-based studies have reported differing associations between HZ and subsequent dementia–some found a positive association and some found an inverse association. In Taiwan, a matched cohort study using the National Health Insurance Research Database found an overall adjusted HR of 1.11 (95% CI 1.04–1.17) [8]; using the same database, a study that focused specifically on herpes zoster opthalmicus reported an HR of 2.83 (95% CI 1.83–4.37) [9]. A large cohort study in South Korea based on the National Health Insurance Service-National Sample Cohort found an overall adjusted HR of 1.12 (95% CI 1.05–1.19) for the association between HZ diagnosis and dementia [10]. In contrast, a case-control study in the same South Korean National Sample Cohort reported an inverse association (OR 0.91, 95% CI 0.84–0.97) ([16] as did a matched cohort study in linked Danish national registries (HR = 0.93, 95% CI 0.90–0.95) [17]. A two-sample Mendelian randomization study using data from the International Genomics of Alzheimer’s Project and the U.K. Biobank reported no clear evidence to suggest an effect of HZ on Alzheimer’s Disease, as the two samples produced differing results (OR = 0.87, 95% CI 0.78–0.96 compared to OR = 1.05, (95% CI 0.995–1.10) [18]. The variation in effect size and direction of the HZ/dementia association in the above studies could be due to differing study designs, definitions of HZ and dementia, statistical methods, and possible bias in documentation of HZ in the medical records.

Both our study and the Bae et al. study [10] found that about 82%—85% of subjects with HZ received antiherpetic medication, whereas the Chen et al. study [8] reported antiherpetic drug use in only 5 percent. Both the Bae et al. and Chen et al. studies examined the association between dementia and any antiherpetic medication use (yes/no) among subjects with HZ. The Bae et al. study found an adjusted HR of 0.79 (95% CI 0.69–0.90 [10] and a HR of 0.77 (95% CI 0.64–0.91 after propensity score matching), while the Chen et al. study reported an adjusted HR of 0.55 (95% CI 0.40–0.77) [8]. Our study found a suggestive inverse adjusted association between antiherpetic medication use and dementia (HR = 0.88, 95% CI 0.77–1.00) with an HR that was closer to the null than those two studies. Another recent publication, Schnier et al, examined the association between antiherpetic medication and dementia in four large European observational cohorts [19]. The reference group for three of the cohorts was people without HZ or antiherpetic treatment. In the one cohort (Denmark) where the authors compared treated to non-treated HZ cases, the HR was 0.90 (95% CI 0.88–0.93), similar to our study’s HR. No trend was observed with number of treatments (1, 2, or 3 or more). Similarly, our study tested for but did not find a trend for either duration or cumulative dose. Neither the Bae nor the Chen studies reported duration or cumulative dose trend analysis.

Several biologic mechanisms for how VZV infection might cause dementia have been proposed. Some of these include VZV leading to increased production of amyloid plaques either directly or through binding to insulin-degrading enzyme, subclinical inflammation of the central nervous system due to VZV invasion, VZV-induced vasculopathy, and induction of systemic inflammatory cytokines [8]. Polymorphisms in the apolipoprotein E gene, APOE, associated with both infectious disease susceptibility and dementia risk, could play a role [3]. By reducing inflammation, antiviral drugs could forestall the long-term adverse effects of HZ [8]. Further research is needed to elucidate these relationships.

The strengths of our study include access to many years of detailed electronic medical records, our study’s large size, the inclusion of both men and women, and the fact that the study was population-based. The comprehensive medical records allowed us to evaluate important potential confounders such as immunosuppression, stroke, and numerous other medical conditions. However, there were important limitations. We used diagnosis codes to define HZ exposure and dementia; the definition of dementia we used (2 diagnosis codes in a 12-month period) was validated in the electronic medical records of a nearby KP region [11]. We identified exposure to HZ as the presence of a HZ diagnosis code in the medical record during the study period, but some subjects with HZ may not have had a medical encounter or may have had an episode of HZ before the study period. We did not employ a lag period for HZ or antiviral use in our primary analysis; however, the proportion with at least one year between HZ and dementia diagnoses was 92%, and we conducted a sensitivity analysis with a 5-year lag period. Although over 93% of HZ-exposed subjects who received anti-herpetic medication had a first prescription date within one week of HZ diagnosis, survival analysis effect estimates for anti-herpetic use may have been slightly biased toward the null by calculating from the HZ diagnosis date rather than the prescription date. The HZ diagnosis codes in the electronic medical records may not all reflect incident HZ infections; some may have been related to history of HZ, although we excluded the code for post-herpetic neuralgia, which can continue long after the HZ infection is resolved. We did not calculate negative predictive value for our HZ definition in this population. As stated by JG Donahue et al [20], ‘negative predictive value is likely to be high given the relatively low prevalence of the disease’. We used diagnosis codes to define the clinical covariates, including hyperlipidemia and hypertension; as with the herpes zoster and dementia definitions reliance on diagnosis codes may have resulted in under-ascertainment of these conditions.

We could not examine individual anti-herpetic medications other than acyclovir, as almost all prescriptions were for acyclovir. For the acyclovir cumulative dose and duration analyses, our analysis was necessarily restricted to filled orders for medication; we were not able to determine whether all the medication was taken. Because many subjects with dementia had ICD-CM-9 or ICD-CM-10 dementia codes that were non-specific, we could not examine subcategories of dementia, such as Alzheimer’s Disease or vascular dementia. We did not have information on some other risk factors for dementia, such as education.

In conclusion, we found little evidence for an association between HZ diagnosis and dementia in the overall population, though women may have slightly elevated risk a few years after the HZ episode. Antiherpetic medication prescribed around the time of HZ diagnosis was statistically associated with lower risk of subsequent dementia in some but not all analyses and subgroups. Further research is warranted to understand the differences in the findings of the various studies.

## Supporting information

S1 Table ICD-9-CM and ICD-10-CM codes for dementia. (DOCX)

S2 Table Herpes zoster diagnosis in relation to hazard of dementia, overall and by age group and sex applying minimum 5-year lag between exposure and outcome. (DOCX)
