# Supplementary material for: Herpes zoster diagnosis and treatment in relation to incident dementia: A population-based retrospective matched cohort study
Source: PLoS One. 2024 Jan 25;19(1):e0296957. doi: 10.1371/journal.pone.0296957 (PMC10810473; doi:10.1371/journal.pone.0296957)
Supplement: S1 Table — (DOCX) [file pone.0296957.s001.docx]

**Supplemental material - 1**

Table S1: ICD-9-CM and ICD-10-CM codes for dementia

| **ICD9 codes** | | | |
| --- | --- | --- | --- |
| 290.0 | 290.10 | 290.11 | 290.12 |
| 290.13 | 290.20 | 290.21 | 290.3 |
| 290.40 | 290.41 | 290.42 | 290.43 |
| 294.0 | 294.10 | 294.11 | 294.20 |
| 294.21 | 294.8 | 331.0 | 331.11 |
| 331.19 | 331.2 | 331.7 | 797 |
| **ICD10 codes** | | | |
| F01 | F02 | F03 | F04 |
| F05 | F06.1 | F06.8 | G13.8 |
| G30 | G30.0 | G30.1 | G30.8 |
| G30.9 | G31.01 | G31.09 | G31.1 |
| G31.2 | G31.83 | G91.4 | G94 |
| R41.81 | R54 |  |  |
