# Supplementary material for: Herpes zoster diagnosis and treatment in relation to incident dementia: A population-based retrospective matched cohort study
Source: PLoS One. 2024 Jan 25;19(1):e0296957. doi: 10.1371/journal.pone.0296957 (PMC10810473; doi:10.1371/journal.pone.0296957)
Supplement: S2 Table — (DOCX) [file pone.0296957.s002.docx]

**Supplemental material - 2**

Table S2 : Herpes zoster diagnosis in relation to hazard of dementia, overall and by age group and sex applying minimum 5-year lag between exposure and outcome

| Variable | No. subjects with dementia/  Total subjects | HR (95% CI) |  |
| --- | --- | --- | --- |
|  |  |  |  |
| Exposed – univariate model | 2,924/96,644 | 1.16 (1.07-1.25) |  |
| Exposed – adjusted for demographics and health care utilization* | 2,924/96,644 | 1.06 (0.98-1.15) |  |
| Exposed – fully adjusted model** | 2,924/96,644 | 1.07 (0.99-1.17) |  |
|  | | | |
| 50 ≤ Age < 65 years** | 428/52,157 | 1.10 (0.88-1.37) |  |
| 65 ≤ Age < 75 years** | 1,938/25,627 | 1.16 (1.05-1.29) |  |
| Age ≥ 75 years** | 2,084 /14,766 | 1.08 (0.99-1.19) |  |
|  | | | |
| Female** | 1,866/59,238 | 1.12 (1.01-1.24) |  |
| Male** | 1,038/37,406 | 0.99 (0.86-1.15) |  |

* Adjusted for age (continuous); gender; race (Black, Asian, White); Hispanic ethnicity; general practice, specialty, and inpatient visits

** Adjusted for age (continuous); gender; race (Black, Asian, White); Hispanic ethnicity; general practice, specialty, and inpatient visits; autoimmune disorder; immunosuppression status; alcohol use disorder; blood disorder; cancer; depression; diabetes; hearing loss; heart failure; hyperlipidemia; hypertension; stroke/TIA; traumatic brain injury; and ever-smoker).
